# Irisin attenuates astrocyte activation and rescues memory in Alzheimer model mice

**DOI:** 10.1002/alz70855_104063

**Published:** 2025-12-24

**Authors:** Luana Heimfarth, Felipe C. Ribeiro, Juliana T S Fortuna, Gustavo Trevizani Stelzer, Sergio T. Ferreira

**Affiliations:** ^1^ Federal University of Rio de Janeiro, Rio de Janeiro, Rio de Janeiro, Brazil

## Abstract

**Background:**

Alzheimer's disease (AD) is a progressive neurodegenerative disease characterized by synapse and memory failure, and severe cognitive impairment. Physical exercise stimulates neuroprotective pathways, has pro‐cognitive actions, and has been reported to alleviate memory impairment in AD. Irisin, an exercise‐induced hormone, is secreted following proteolytic cleavage of fibronectin type‐III‐domain‐containing 5 (FNDC5). Irisin regulates peripheral metabolism, and has been found to protect synapses and rescue memory in mouse models of AD. The aim of the current study was to evaluate the possible role of irisin in astrocytes within the framework of AD pathophysiology.

**Methods:**

We exposed primary cortical astrocyte cultures to irisin (25 nM), and the expression of neurotrophic factors, α5β5 integrin receptor, and LDH5α were determined by RT‐PCR. MAPK activity was determined by Western blot. Cognitive impairment was investigated in C57BL/6 mice that received an intracerebroventricular infusion of Aβ oligomers (AβOs, 10 pmol) in the presence or absence of irisin (30 pmol). The expression of neurotrophic factors was determined in the hippocampus and frontal cortex of the same animals 7 days after the AβOs or irisin infusion by RT‐PCR.

**Result:**

We demonstrate that irisin promotes the expression of brain‐derived neurotrophic factor (BDNF), nerve growth factor (NGF), glial cell line‐derived neurotrophic factor (GDNF), α5 subunits of integrin receptors and LDH5α, and stimulates transient activation of ERK 1/2 in astrocytes. We further show that irisin attenuates memory impairments in an AD mouse model, and increases hippocampal expression of BDNF, GDNF and NGF *in vivo*.

**Conclusion:**

Our findings support the idea that physiological protection by irisin against synaptotoxic AβOs can be mediated by modulation of astrocyte‐derived factors.

